# Leave or Stay as a Risky Choice: Effects of Salary Reference Points and Anchors on Turnover Intention

**DOI:** 10.3389/fpsyg.2018.00686

**Published:** 2018-05-18

**Authors:** Guanxing Xiong, X. T. Wang, Aimei Li

**Affiliations:** ^1^School of Economics and Management, South China Normal University, Guangzhou, China; ^2^Key Lab for Behavioral Economic Science & Technology, South China Normal University, Guangzhou, China; ^3^School of Humanities and Social Science, The Chinese University of Hong Kong, Shenzhen, China; ^4^Psychology Department, University of South Dakota, Vermillion, SD, United States; ^5^School of Management, Jinan University, Guangzhou, China

**Keywords:** reference points, anchors, turnover intention, risky choice, minimum requirement, salary goal, pay satisfaction

## Abstract

Within a risky choice framework, we examine how multiple reference points and anchors regulate pay perception and turnover intentions in real organizational contexts with actual employees. We hypothesize that the salary range is psychologically demarcated by three reference points into four regions, the minimum requirement (MR), the status quo (SQ), and the goal (G). Three studies were conducted: Study 1 analyzed the relationship between turnover intention and the subjective likelihood of falling into each of four expected salary regions; Study 2 tested the mediating effect of pay satisfaction on salary reference point-dependent turnover intention; and Study 3 explored the anchoring effect of estimated peer salaries. The results show that turnover intention was higher in the region below MR or between SQ and G but lower in the region above G or between MR and SQ. That is, turnover intention can be high even in situations of salary raise, if the raise is below a salary goal (i.e., leaving for a lack of opportunity) and low even in situations of salary loss, if the expected salary is still above the MR (i.e., staying for security). In addition, turnover intention was regulated by pay satisfaction and peer salaries. In conclusion, turnover intention can be viewed as a risky choice adapted to salary reference points.

## Introduction

Turnover in organizations has been a central topic for economists, psychologists, and management scholars. March and Simon’s (1958) work marked the beginning of the attempt to develop an overall theory explaining why people leave their jobs (Mobley et al., 1979). However, the main stream of turnover research has been focused on job satisfaction and dissatisfaction (see Tett and Meyer, 1993; Griffeth et al., 2000; for reviews). More recently, some researchers have re-emphasized the value of studying turnover as a risky choice (i.e., Allen et al., 2007). From a decision-making perspective, Steel (2002) views turnover as an employee-driven process of opportunity search and goal pursuit beyond job satisfaction or dissatisfaction. Similarly, Direnzo and Greenhaus (2011) emphasize the active and voluntary nature of turnover in the boundaryless world of organizations.

The development of the behavioral decision-making research over the last two decades has provided us with a useful framework to better understand and predict actual life choices. In the present studies, we examine how multiple salary reference points and anchors regulate pay perception and turnover intention in organizational contexts. When making job-related decisions, individuals simultaneously consider their salary bottom line, current pay level, and desired salary (Wang and Johnson, 2012). These salary reference points can also be regulated by peer comparison. Employees are often more focused on relative salary differences than absolute differences; for example, a meta-analysis of 203 studies revealed that discrepancy between actual pay and deserved pay is the primary determinant of turnover intention and actual turnover (Williams et al., 2006). As suggested by equity theory and the discrepancy models of pay satisfaction (Festinger, 1954; Buunk and Gibbons, 2007), we assume that employees take the initiative to compare themselves to others within or outside the current organization, and the results of such comparisons influence employee turnover intention.

### Turnover as a Risky Choice

One aspect of turnover decision that has not been adequately considered in the extant turnover models is the risk associated with quitting one’s job (Allen et al., 2007; Vardaman et al., 2008). We view turnover decision as a risky choice driven by perceived job security and opportunity. In the fields of behavioral decision-making and finance, risk is typically defined as the variance in expected outcomes (Markowitz, 1952). In an organization, employees’ turnover choices can be either risk-seeking or risk-averse. A key factor that determines turnover as being risk-seeking or risk-averse is whether the employee has an alternative job offer in hand (e.g., Michaels and Spector, 1982; Lee and Mitchell, 1994). With a specific alternative offer in hand, the expected salary of staying with the current organization may be more variable than the sure alternative offer. For example, for an employee of a small high-technology start-up firm who is offered a higher paying job with a large, established company, staying might be a riskier option. In contrast, when considering turnover without a certain alternative offer, as examined in the current studies, staying would be less risky than leaving (Maccrimmon and Wehrung, 1985). A higher variance in the expected outcomes associated with a new job (i.e., it may be better or worse than the current one) makes turnover a risky choice. Since a new job is often associated with a higher social and organizational uncertainty and higher cultural and personal unfamiliarity, quitting a currently held job is thus generally considered riskier than staying with the current job.

Turnover intention thus is not only a result of passive reaction due to job dissatisfaction but also involves active evaluations of job security and opportunity. Risk taking of managers are actively regulated by their perceived occupational rewards (Ceschi et al., 2017). An active job search for better opportunities has become a normalcy for employees in today’s world of boundaryless workplaces (Direnzo and Greenhaus, 2011). Boswell et al. (2005) showed that turnover decisions are made based on risk perception involving both positive and negative expectations. Such expectations are often derived from comparisons against decision reference points (e.g., salary goal or minimum requirement) and decision anchors (e.g., the pay levels of peers). Risk perception in job-related contexts is affected by perceived organizational security or insecurity (Weng and Mcelroy, 2012) and opportunities (Sverke et al., 2002; Staufenbiel and König, 2010). Such active evaluations of job security and opportunities would either evoke or inhibit turnover intention, which in turn determines actual job turnover in organizations (e.g., Tett and Meyer, 1993).

One of the key factors that determines perceived job security and opportunity, and consequently turnover decisions, is salary amount (Jurgensen, 1978; Cotton and Tuttle, 1986; Cable and Judge, 1994; Burchell et al., 2002; Salleh and Memon, 2015). Pay satisfaction is largely determined by the discrepancies between actual salary and personal salary reference points, such as what employees feel they deserve, want, or see others receiving (Goodman, 1974; Lawler, 1981).

Interestingly, pay-satisfaction research shows that despite the importance of pay, the correlation between how much people are paid and their satisfaction with their pay is only modest. The results of a meta-analysis show that actual salary generally accounts for less than 23% of the variance in pay satisfaction, and absolute pay is only marginally related to pay satisfaction (Judge et al., 2010). The present study extends the previous research on turnover determinants by identifying two common sources of job dissatisfaction: failure to reach a salary goal, and failure to maintain distance from a minimum salary requirement. Therefore, the relationship should be regulated by not only actual pay but also three pay-related reference points: the expected salary, the desired salary, and the minimum acceptable salary.

### Tri-Reference Point Theory and Turnover Analysis

**FIGURE 1:**
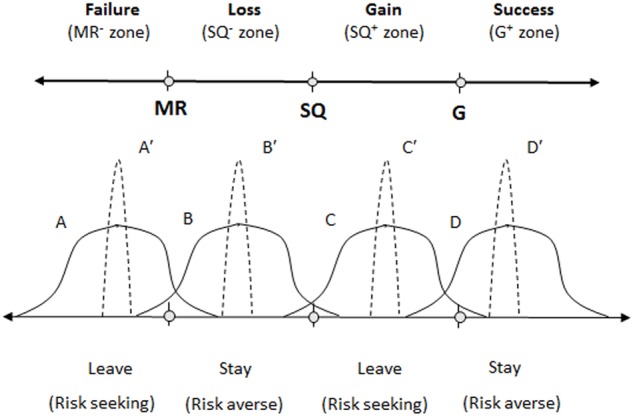
Expected salary outcome regions demarcated by three reference points and predicted choice preference to leave or stay. MR, minimum requirement; SQ, status quo; G, goal. A *versus* A′, B *versus* B′, C *versus* C′, and D *versus* D′ represent low-variance option *versus* high-variance option of the same expected value, respectively.

Recent developments in the field of behavioral decision-making suggest that individuals in various risky choice situations use multiple reference points to guide their decision-making. Based on the tri-reference point (TRP) theory of risky choice (Wang and Johnson, 2012), decision makers strive to reach a goal and at the same time avoid falling below a bottom line. Prospect theory (Kahneman and Tversky, 1979) demonstrates that the carrier of subjective value is not the total wealth but changes from the status quo (SQ) that separate choice outcomes into gains and losses. The TRP theory extends prospect theory by further dividing expected outcome space into four functional regions: negative outcomes are divided into failure and loss regions separated by the minimum requirement (MR) reference point, while positive outcomes are divided into gain and success regions separated by the goal (G) reference point (see the upper panel of **Figure 1**).

Tri-reference point theory assumes that the settings of MR and G can be either externally given or internally generated, and they are fine-tuned by the task environment (e.g., peer salary). These reference points effectively divide the outcome space into four distinct regions: failure (MR^-^ zone), loss (SQ^-^ zone), gain (SQ^+^ zone), and success (G^+^ zone).

In the context of pay in organizations, the minimum salary required can be viewed as an employee’s MR; the G is an employee’s desired salary; and the SQ is his or her actual salary. Implied in job-satisfaction-based turnover models, pay levels are expected to be linearly or semi-linearly related to turnover intention (e.g., Griffeth et al., 2000; Judge et al., 2010). In other words, increasing pay should decrease turnover intention and never increase turnover intention, and vice versa. In contrast, as illustrated in the lower panel of **Figure 1**, it is predicted that turnover intention is non-linearly related to pay levels.

Along the outcome value dimension, high-variance options (A, B, C, and D) and low-variance options (A′, B′, C′, and D′) are paired, presenting four independent choice tasks, respectively, see in **Figure 1**. Imagine that these options represent different salary distributions. When making a choice between the risky option A and the safer option A′, TRP theory predicts a strong risk-seeking preference for A because it offers the only chance of staying above the MR. According to the TRP theory, MR has a higher priority than G in determining choice preference, thus, when encountering a choice between B and B′, we predict a strong risk-averse preference for the safe option B′ because the possibility of falling below MR outweighs the potential gain (above SQ). Similarly, a weak risk-seeking preference for C over C′ is expected because the small chance of being above G would be valued more highly than would the small probability of falling below the SQ due to the TRP priority order of MR > G > SQ, and a risk-averse preference for D′ is expected because the safer option D′ is well above the goal. A general mean-variance principle can be derived from the above analysis: be risk- or variance-seeking when the expected value of choice outcome is below MR (or G); however, be risk- or variance-averse when the expected value is above MR (or G).

### Current Approach and Hypotheses

Pay changes are common in organizations; employees often experience and anticipate pay changes (e.g., salary reform, raises, and pay cuts). Thus, most of the time, an employee’s expected salary SQ varies between the G and the MR. This assumption excludes situations of SQ < MR (i.e., individual’s expected salary SQ below his or her salary minimum requirement) or SQ > G (i.e., individual’s expected salary SQ above his or her salary goal) from our present analysis, as these two situations are not very common in organizations. When making a turnover-related decision, an employee anticipates what his or her expected salary will be if he or she stays with the same job in reference to his or her salary G, SQ, and MR. These salary reference points are situational and regularly updated as a result of changes in job status and work environment. Notably, to focus on the most accessible and available comparisons in a turnover-related process, we measured only the expected salary in the current company instead of the expected salary of an alternative offer.

As shown in **Table 1**, the region below MR (MR^-^ zone, a failure region) indicates that an employee who is expecting an unacceptable salary situation, is likely to leave the organization since the expected salary is already below the MR. This is thus a region of “leaving for lack of security” since employees will feel insecure living with a salary that is below their minimal requirement.

**Table 1:** Predicted risk-preference and turnover intention based on employee expected salary change falling into each of the four reference-point demarcated outcome regions *versus* pay-satisfaction-based predictions.

Expected salary change	Satisfaction-based predictions	Reference points dependent predictions
MR^-^ zone (Failure) Expected salary decrease	Dissatisfaction Leaving	Risk (variance) seeking Leaving for lack of security Reluctant leavers
SQ^-^ zone (Loss) Expected salary decrease	Dissatisfaction Leaving	Risk (variance) averse Staying for security Reluctant stayers
SQ^+^ zone (Gain) Expected salary increase	Satisfaction Staying	Risk (variance) seeking Leaving for lack of opportunity Enthusiastic leavers
G^+^ zone (Success) Expected salary increase	Satisfaction Staying	Risk (variance) averse Staying for opportunity Enthusiastic stayers

Second, the region between MR and SQ (SQ^-^ zone, a loss region) indicates that an employee who is expecting a future salary in this region is unlikely to leave the organization since the expected salary is above his or her MR. This is thus a region of “staying for security” since higher variance of alternative jobs may fall below the salary MR.

Third, the region between SQ and G (SQ^+^ zone, a gain region) indicates that an employee who is expecting a future salary in this region is likely to leave the current organization since the expected salary for staying is below his or her salary goal. This is thus a region of “leaving for lack of opportunity” since an expected pay raise is not enough to reach the salary G.

Fourth, the region above G (G^+^ zone, a success region) indicates that an employee who is expecting a future salary in this region is unlikely to leave the current organization because the expected salary can exceed his or her salary goal if he or she stays with the same company. This is thus a region of “staying for opportunity.”

Hom et al. (2012) view employee turnover as a decision-making process and focus on the motivational states that precede a turnover choice of leave or stay. They identified two overarching dimensions underlying the motivational states leading to turnover decisions: desired employment status (whether employees want to stay or leave) and perceived volitional control due to job-market demand and available alternatives (see also Wheeler et al., 2013). Crossing these dimensions yields four prime states and thus four types of turnover situations: enthusiastic leavers, reluctant leavers, enthusiastic stayers, and reluctant stayers. As shown in **Table 1**, these four types of turnover situations are consistent with the TRP-classification of the turnover situations corresponding to each of the four functional regions of expected salary outcomes.

Based on the above theoretical analyses, we predict the following:

*Hypothesis 1*. Turnover intention will be higher when the expected salary of the current job falls into the two non-adjacent risk-seeking regions: the MR^-^ zone associated with “leaving for lack of security,” and the SQ^+^ zone associated with “leaving for lack of opportunity.” In contrast, turnover intention will be lower when the expected salary falls into the two non-adjacent risk-averse regions: the SQ^-^ zone associated with “staying for security,” and the G^+^ zone associated with “staying for opportunity.”

These predictions contrast with those based on job satisfaction or dissatisfaction (see **Table 1**). In other words, rather than simply being dissatisfied with his or her pay, an employee who anticipates a pay cut may be either risk-seeking for alternative jobs if the expected salary falls below his MR, or risk-averse to staying if the expected salary is below his or her salary SQ but above the MR.

As employee’s salary SQ varies between the G and the MR. Specifically, we predict that the salary distance between SQ and MR (SQ-MR distance) and between SQ and G (G-SQ distance) would be used to make turnover-related decisions. In addition, when (G – SQ) > (SQ – MR) such that SQ is closer to MR than to G, we predict that an employee would have higher turnover intention to avoid the risk of falling below the MR. Second, when (G – SQ) < (SQ – MR) such that SQ is closer to G than MR, we predict that an employee will have lower turnover intention to obtain a more achievable G within the current organization. We further predict that pay satisfaction reflects and thus mediates the perceived distances between the three salary reference points.

*Hypothesis 2*. Pay satisfaction mediates the relationship between SQ-MR (G-SQ) distance and turnover intention.

In the present studies, we also examined how salary G and MR could be affected by peer comparison (e.g., Festinger, 1954; Buunk and Gibbons, 2007). Peer salary is expected to regulate the settings of salary G and MR as an anchor (Tversky and Kahneman, 1974) and subsequently affect turnover intention.

*Hypothesis 3*. When estimated peer salary > employee own expected salary (SQ), the setting of the salary G and MR will be upregulated and thus increase turnover intention. When estimated peer salary < expected own salary (SQ), the setting of the salary G and MR will be downregulated and thus lower turnover intention.

In the following three studies, the above three hypotheses were tested with independent samples of actual employees from different companies. None of the participants had received alternative job offers at the time of participation. Studies 1, 2, and 3 were designed to test Hypotheses 1, 2, and 3, respectively.

## Study 1

As shown in **Figure 1** and **Table 1**, the two outcome regions (zones) above the SQ represent satisfaction regions, and the two zones below the SQ represent dissatisfaction regions. The job-satisfaction-based hypothesis predicts that turnover intention is positively correlated with the subjective likelihood of falling into the two dissatisfaction regions (MR^-^ and SQ^-^ zones), and it is negatively correlated with the subjective likelihood of falling into the two satisfaction regions (SQ^+^ and G^+^ zones). In contrast, Hypothesis 1 predicts that turnover intention is positively correlated with the subjective likelihood of falling into the two non-adjacent risk-seeking regions (MR^-^ and SQ^+^ zones), and it is negatively correlated with the subjective likelihood of falling into the two non-adjacent risk-averse regions (SQ^-^ and G^+^ zones). In other words, increasing pay can either reduce or increase turnover intention.

### Method

#### Participants

One hundred and seventeen full-time employees (71 men and 46 women; *M_age_* = 26.4 years, *SD* = 1.38) were recruited in Study 1. The participants were part-time students taking courses in a management program at a large university in Guangzhou, China. In this sample, 68% of the participants were married; 57% of the participants were operational staff who held non-managerial positions; 31% were junior managers; and 12% were middle/senior managers (see also **Table 2**).

**Table 2:** Means, standard deviations, and Pearson correlations of the measured variables (*N* = 117).

Variable	*M*	*SD*	1	2	3	4	5	6	7	8	9
(1) Gender (0 = male; 1 = female)	0.39	0.49	–								
(2) Age	26.4	1.38	–0.21*	–							
(3) Marital status (0 = unmarried; 1 = married)	0.32	0.47	0.28**	0.27**	–						
(4) Post-level (1 = general staff; 2 = junior manager; 3 = senior manager)	1.55	0.70	–0.13	0.25**	0.05	–					
(5) Subjective likelihood of falling into dissatisfaction zones (MR^-^ and SQ^-^)	13.25	11.74	0.01	–0.16	–0.10	–0.17	–				
(6) Subjective likelihood of falling into satisfaction zones (SQ^+^ and G^+^)	36.74	11.74	–0.01	0.16	0.10	0.17	–1.00**	–			
(7) Subjective likelihood of falling into risk-seeking zones (MR^-^ and SQ^+^)	27.40	10.40	–0.17	0.15	0.21*	–0.08	–0.17	0.17	–		
(8) Subjective likelihood of falling into risk-averse zones (SQ^-^ and G^+^)	22.60	10.40	0.17	–0.15	–0.21	0.08	0.17	–0.17	–1.00**	–	
(9) Turnover intention	2.82	0.68	–0.10	–0.07	–0.09	–0.23*	0.03	–0.03	0.23*	–0.23*	–

*^∗^*p* < 0.05; ^∗∗^*p* < 0.01.*

#### Procedure

**FIGURE 2:**
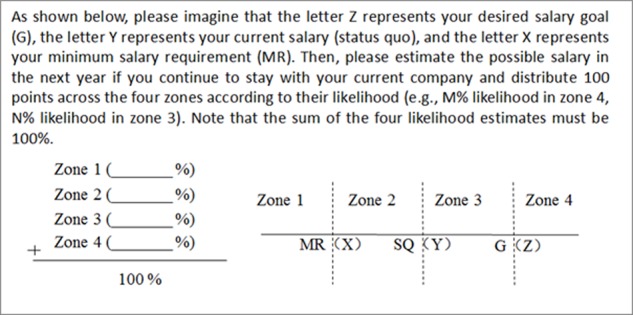
Expected salary distribution task.

The participants were asked to estimate their expected salary for the next year in terms of the likelihood of it falling into each of the four possible salary zones. The expected salary next year can be either a pay raise or a pay cut. See **Figure 2** for a detailed description of the expected salary distribution task. In this task, the current salary was the SQ salary, the goal salary was the desired and the achievable salary, and the salary MR was the minimum salary requirement.

Next, the participants were asked to rate items on a five-point Likert-type turnover intention scale (from 1 = *“Definitely No”* to 5 = *“Definitely Yes”*), which was adopted from Farh et al. (1998). The scale consisted of four items (α = 0.84) that ask about an individual’s intention to leave his or her job. For example, the first item was “I frequently think of quitting my job.” The participant’s turnover intention was measured as the average of the item scores.

### Results and Discussion

**FIGURE 3:**
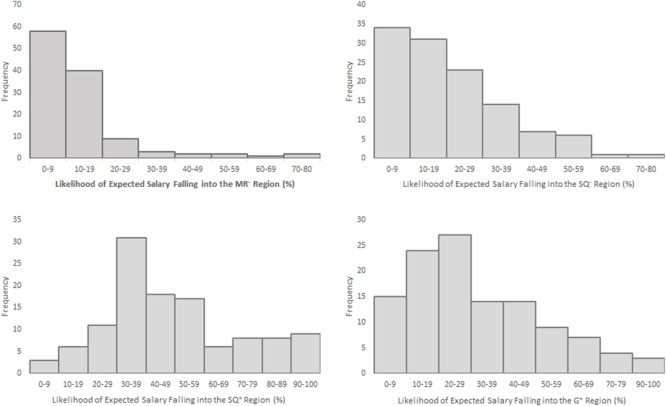
Histograms of likelihood estimates of expected salary falling into the MR^-^, SQ^-^, SQ^+^, and G^+^ zones in Study 1. MR^-^ zone: expected salary after a pay cut was below MR; SQ^-^ zone: expected salary after a pay cut was above MR and below SQ; SQ^+^ zone: expected pay raise was above SQ and below G; G^+^ zone: expected pay raise was above G.

**Figure 3** shows frequency data of the likelihood estimates of expected salary falling into the MR^-^ zone, SQ^-^ zone, SQ^+^ zone, and G^+^ zone. We conducted separate regression analyses to evaluate the predictions based on the job-satisfaction hypothesis and the predictions derived from Hypothesis 1. **Table 2** shows the means, standard deviations, and correlations of the measured variables.

Consistent with Hypothesis 1 (see in **Table 3**), turnover intention was positively correlated with the subjective likelihood of falling into the risk-seeking zones (MR^-^ and SQ^+^) (γ_(MR- and SQ+)_= 0.23, *p* < 0.05) and negatively correlated with the subjective likelihood of falling into the risk-averse zones (SQ^-^ and G^+^) (γ_(SQ- and G+)_= -0.23, *p* < 0.05). In contrast, turnover intention was not significantly related to the subjective likelihood of falling into either the dissatisfaction zones (MR^-^ and SQ^-^) or the satisfaction zones (SQ^+^ and G^+^) (γ_(MR- and SQ-)_= 0.03, γ_(SQ+and G+)_= -0.03; both *p* > 0.05). Note that the satisfaction zones and dissatisfaction zones were separated based on the assumption that employees would be more satisfied with pay raises (above salary SQ) and dissatisfied with pay cuts (below salary SQ).

**Table 3:** Regression analysis of the relationships among demographical variables, job-related variables, expected salary distribution scores, and turnover intention (*N* = 117).

Variable	Control model	Satisfaction-dependent model		Tri-reference point dependent model	
		Zone MR^-^ and SQ^-^	Zone SQ^+^ and G^+^	Zone MR^-^ and SQ^+^	Zone SQ^-^ and G^+^
Gender (0 = male; 1 = female)	–0.13	–0.13	–0.13	–0.07	–0.07
Age	–0.03	–0.04	–0.04	–0.05	–0.05
Marital status (0 = unmarried; 1 = married)	–0.03	–0.03	–0.03	–0.09	–0.09
Post-level (1 = general staff; 2 = junior manager; 3 = senior manager)	–0.24*	–0.24*	–0.24*	–0.21*	–0.21*
Subjective likelihood of falling into dissatisfaction zones (MR^-^ and SQ^-^)		–0.03			
Subjective likelihood of falling into satisfaction zones (SQ^+^ and G^+^)			0.03		
Subjective likelihood of falling into risk-seeking zones (MR^-^ and SQ^+^)				0.23*	
Subjective likelihood of falling into risk-averse zones (SQ^-^ and G^+^)					**-**0.23*
Model summary					
*R*^2^	0.073	0.074	0.074	0.118	0.118
Δ*R*^2^		0.001	0.001	0.045*	0.045*
*F*	2.21	1.76	1.76	2.98	2.98
Probability	0.07	0.13	0.13	0.02*	0.02*

*^∗^*p* < 0.05; ^∗∗^*p* < 0.01.*

We further analyzed the relationship between turnover intention and the subjective likelihood of falling into a single zone. The results showed that turnover intention was positively correlated with the subjective likelihood of falling into the SQ^+^ (leaving for opportunity) zone (γ_(SQ+)_ = 0.19, *p* < 0.05), and it was negatively correlated with the subjective likelihood of falling into the G^+^ (staying for opportunity) zone (γ_(G+)_ = -0.24, *p* < 0.05). This result indicates that an expected pay raise can either increase or decrease turnover intention. However, the turnover intention scores were not significantly correlated with the subjective likelihood of falling into either the MR^-^ (leaving for lack of security) zone or the SQ^-^ zone (staying for security), γ_(_*_MR_*_-)_ = 0.12, γ_(_*_SQ_*_-)_ = -0.003. This lack of significant correlations is likely a result of low frequencies in these two salary regions. Most participants thought it was unlikely that they would experience a pay cut (see **Figure 3**). In other words, the participants were unlikely to react to situations that were unlikely to occur.

We adopted a subtraction method, using a dependent measure in gains minus that in losses (Levin and Hart, 2003). Specifically, we used probability allocation scores in the domain of gain (SQ^+^ plus G^+^) minus the probability allocation scores in the domain of loss (SQ^-^ plus MR^-^) to test the prediction that the difference in the likelihood allocation scores between the two risk-averse regions (SQ^-^ plus G^+^) and the two risk-seeking regions (MR^-^ plus SQ^+^) should be negatively correlated with turnover intention. As predicted, the difference score was significantly and negatively correlated with turnover intention (*r* = -0.23, *p* = 0.01, two-tailed).

## Study 2

In Study 2, we further tested Hypothesis 2 against the pay-satisfaction-based predictions. Using the SQ as the cutting point, a salary dimension from low to high can be divided into regions of dissatisfaction and satisfaction. We tested the prediction that pay satisfaction reflects the discrepancy between the salary MR and SQ (or its complementary discrepancy between the salary G and SQ) and mediates the discrepancy effect on turnover intention.

### Method

**FIGURE 4:**
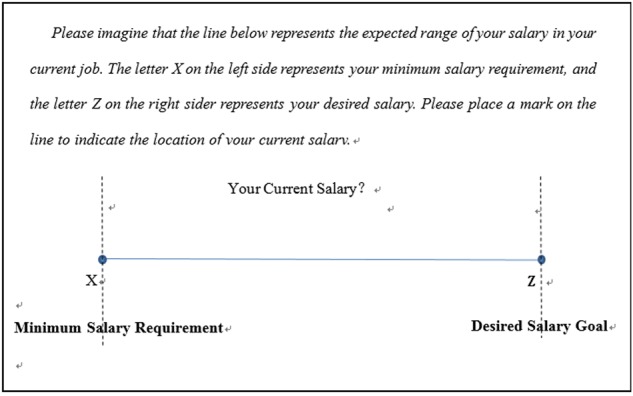
An analog scale of subjective pay discrepancies. Participants estimate and locate on the scale their salary status quo in relation to their salary goal and minimum requirement.

We recruited 68 full-time employees (43 females; *M_age_* = 28.4 years, *SD* = 4.22) who were taking part-time courses in an MBA program at a large university in Guangzhou, China. We developed an analog version of the salary discrepancy scale (see **Figure 4**), which was based on the methods used in previous studies (Scholl et al., 1987; Blau, 1994). This analog scale of salary discrepancies simultaneously takes into consideration three reference points (i.e., salary SQ, MR, and G).

In a pre-test, we surveyed a group of 100 working people (50 men and 50 women). Only four participants (4%) considered their salary SQ to be either below MR or above G. Based on this result, the salary range used in the study excluded the exceptional cases of SQ < MR or SQ > G.

The participants were asked to place a mark on a 100-mm line segment to indicate the location of their salary SQ in relation to their salary G and MR, with the left and right endpoints of the line representing salaries MR and G, respectively. The distance in millimeters from the mark to the left end of the line segment was recorded as the salary SQ-MR discrepancy; and the discrepancy in millimeters from the mark to the right end of the line segment was recorded as the salary SQ-G discrepancy. The participants were then asked to complete the same turnover intention questions used in Study 1.

In addition, they rated their pay satisfaction on a single-item five-point scale, where five stood for highly satisfied and one for highly dissatisfied (i.e., “How would you rate your degree of satisfaction with your current pay and salary condition?”). The use of a single-item approach measuring job-related satisfaction has been shown to be highly correlated with multiple-item measures and in some cases accounted for the incremental variance in self-reported intentions to turnover (e.g., Nagy, 2002).

### Results and Discussion

The SQ-G discrepancy was positively correlated with turnover intention. In contrast, the SQ-MR discrepancy was negatively correlated with turnover intention (γ = -0.31, *p* < 0.01). Since the two discrepancy measures were complementary, we only used the SQ-MR discrepancy in the following analysis to test our predictions.

Using linear regression, we compared how pay satisfaction and salary discrepancy, alone and in combinations, predicted turnover intention. The regression beta coefficient without controlling for age and gender was -0.311, and was -0.26 after controlling for age and gender (adjusted *R*^2^ = 0.112, *F* = 3.82, *p* = 0.014).

The results from the bootstrap analysis using the PROCESS macro (Preacher and Hayes, 2008; Hayes, 2013) revealed a full mediation effect of pay satisfaction in predicting turnover intention from expected salary discrepancy (i.e., the SQ-MR distance). This bootstrap method (with 5000 replications) revealed that the 95% confidence interval for the indirect effect of the SQ-MR discrepancy via pay satisfaction was estimated to lie between -0.1353 and -0.0068. Because zero was not in the 95% confidence interval, we can conclude that the indirect effect is significantly different from zero at *p* < 0.05. The direct effect of the SQ-MR discrepancy on turnover intention was no longer significant after controlling for pay satisfaction (*t* = -1.107, *p* = 0.273).

## Study 3

Study 3 tested Hypothesis 3 regarding the effects of estimated peer salary on employee turnover intention with a natural sampling design that classified the actual employees into either the below a hypothetical peer-salary group or the above the peer-salary group.

### Method

We recruited 66 full-time employees (39 females; *M_age_* = 25.3 years, *SD* = 1.44) from different companies who were taking part-time courses in a management program at a large university in Guangzhou, China. The participants were asked to provide their salary SQ (i.e., their current actual monthly salary), their monthly salary G, and their salary MR. The instruction used to get salary G and MR were the same as used in Study 1. In addition, they were asked to estimate the average peer salary of similar companies in Guangzhou, China. This question was used to elicit peer comparison without imposing a salary anchor that is explicitly higher or lower than the participant’s salary SQ. The participants indicated their turnover intention on a seven-point scale that described the possibility of leaving their current job this year. According to the self-salary and the estimated peer salary, the participants were classified into one of two groups: Group 1 consisted of the participants (*N* = 37) whose estimated peer salary > their salary SQ, and Group 2 consisted of the participants (*N* = 29) whose estimated peer salary < their salary SQ.

### Results and Discussion

The setting of salary reference points was measured in terms of the ratios of G/SQ and MR/SQ instead of the absolute values of salary MR and G distance. The absolute measures of MR and G may not be practically adequate in actual organizational situations where the same salary G or MR may have different effects on turnover intention depending on its location relative to the individual’s salary SQ. The advantage of using the ratios of reference points is that the ratios are standardized measures that are independent of individual differences in salary SQ.

Using the ratio of G/SQ as a measure of relative distance to salary G and the ratio of MR/SQ as a measure of relative distance to salary MR, significant differences were found between the two groups of employees whose salary SQ was self-estimated as either above or below their peer salary average. Independent sample *t*-tests were conducted to compare the effects of peer salary on the ratios of G/SQ and MR/SQ and the turnover intention between groups.

Supporting Hypothesis 3 (see in **Table 4**), compared to the employees whose salary SQ was higher than the estimated peer salary at similar companies, the employees with a salary SQ lower than the estimated peer salary had a significantly higher G/SQ [*t*(64) = 2.33, *p* < 0.05] and MR/SQ [*t*(64) = 2.71, *p* < 0.01] and a significantly higher turnover intention [*t*(64) = 2.13, *p* < 0.05]. These results indicate that higher peer salary elevated the setting of salary reference points (both G and MR), whereas lower peer salary reduced the settings of the salary reference points.

**Table 4:** Effects of self-estimated peer salary on the setting of salary G, MR, and turnover intention of employees in Study 3.

Group	*N*	G/SQ ratio (*M* ±*SD*)	MR/SQ ratio (*M* ±*SD*)	Turnover intention (*M* ±*SD*)
Peer salary > SQ	37	1.45 ± 0.25	0.81 ± 0.11	4.30 ± 1.33
Peer salary < SQ	29	1.30 ± 0.23	0.74 ± 0.11	3.66 ± 1.05
*t*-Test		*p* = 0.023	*p* = 0.009	*p* = 0.037

Although subjectively the participants perceived that their current salary was either above or below a peer group (comparable employees in a similar company), the means of the reported salary from the two participant groups (3668 ± 905 vs. 4559 ± 1195 Yuan) did not differ significantly (*t* = -1.083, *p* = 0.283). This result suggests that the effect of estimated peer salary was caused by the discrepancy between the actual salary and a salary reference point instead of the absolute value of the actual salary of the participants.

## Implications, Limitations and Conclusions

### Theoretical Implications

The three studies tested predictions derived from the TRP theory by viewing turnover as a risky choice in organizations. The results challenge the conventional wisdom of turnover intention as a linear function of pay levels and pay satisfaction. Viewing turnover as a reference point-dependent risky choice offers counterintuitive insights for organizational management. A pay raise can increase turnover intention if it is below and close to a personal salary G.

In Study 1, the participants who anticipated a salary raise in the next year but expected it to be lower than their salary goal (SQ^+^ zone) had a higher turnover intention than those who anticipated a pay raise above their salary goal (G^+^ zone). These results shed light on the question “why do some employees who are satisfied with their jobs leave, while many who are dissatisfied stay?” (Allen et al., 2007). The individual differences in salary reference points may also account for individual preference for either fixed salary plans, if the expected salary is in the SQ^-^ or G^+^ zone or variable salary plans, if the expected salary is in the MR^-^ or SQ^+^ zone (Pappas and Flaherty, 2006; Wang and Johnson, 2012).

The results of Study 3 showed that the salary reference points were sensitive to social comparison against estimated peer salary levels. Extending the previous studies on pay dispersion and expected salary gap (e.g., Downes and Choi, 2014), the results revealed that salary anchors in social comparisons affect not only the setting of salary goals and bottom lines but also employees’ turnover intention.

### Managerial Implications

The quit-or-stay decision is ubiquitous in organizations. From a decision-making perspective, organizational turnover should be viewed as more of an employee-driven process rather than a management-driven process. Turnover is ultimately an individual decision on the part of employees. A direct implication for human resource (HR) management from the present studies is that HR managers should regularly update their estimates of the salary goals and bottom lines of prospective employees. The result of a survey given to 63 HR managers conducted by one of the authors in Shanghai, China, a few years ago showed that the HR managers significantly underestimated the salary MR and overestimated the salary G of the reference group.

To update their estimate of employees’ salary Gs and MRs, HR managers should implement annual surveys to ask current and prospective employees about their salary Gs and MRs in the context of career development planning. Since turnover intention tends to peak either when the salary is low and near the MR or when it is high and near the G, more accurate estimates of individual employees’ salary reference points would enable HR managers to develop more individualized plans for the timing and amount of pay changes.

The present results also suggest two types of practical indicators of turnover intention (i.e., the SQ-G and SQ-MR discrepancy and the relative discrepancy reflected by the G/SQ ratio and MR/SQ ratio) for HR management. More importantly, these expected salary ratios predicted turnover intention better than the actual salary of employees (Study 3). These indicators can be used to either predict or evaluate the stability of different employees, different departments within a company, or different companies in the same sector of an industry. In addition, the results of Study 3 suggest that implicit peer comparison can either increase or reduce employees’ turnover intention by changing their perceived distance between the salary SQ and salary G or MR.

Although a pay raise should always be better than a pay cut, turnover intention can still be heightened in situations of pay raise due to the lack of opportunity to reach a higher salary goal. HR management should be based on employees’ updated salary goals and MRs. Traditionally, to proportionally reward good performance, managers will raise the salary of Employee G^+^ more than Employee SQ^+^ if the performance of the former is better than the latter. However, a raise for Employee G^+^ may not be necessary since he or she is already an “enthusiastic stayer.”

In contrast, a larger salary raise may be needed for Employee SQ^+^ who otherwise is potentially an “enthusiastic leaver” (see **Table 1**).

### Limitations

We recognize three limitations of the current studies. First, we focused on the relationships between salary perceptions and turnover intention rather than actual turnover behaviors. Turnover intention may not have a direct linear relationship with actual turnover. Actual turnover is only moderately related to turnover intention (Steel and Ovalle, 1984). Further research should be conducted to investigate the relationships between turnover intention and actual turnover.

Second, our studies are limited to only turnover-related decision reference points and perceived discrepancies, while many other significant factors such as actual performance are left out under the assumption that their effects are controlled by random sampling and random assignments. However, given the relatively small sample sizes constrained by our surveys and experimental designs, these other factors could also affect our data in a covert manner.

Third, our studies have been conducted in China, which may limit the trans-cultural validity of our results.

### Conclusion

Following March and Simon (1958) and in line with more recent efforts to study turnover within a framework of risky choice (e.g., Donnelly and Quirin, 2006), we argue that the mechanism of turnover intention can be analyzed within a framework of reference point-dependent risky choice. The studies reported in this paper tested the validity of this approach and the extent to which turnover intention can be accounted for by reference-point dependent risk taking. The results revealed two triggering conditions of withdrawal cognition: turnover intention tends to increase either when the salary is low and near the salary MR or when it is high and near the salary G. HR management should be based on employees’ updated salary goals and MRs. A turnover decision can be viewed as a risky choice adapted to both internal expectations (salary G or MR) and estimated peer salaries.

## Ethics Statement

The study was approved by the ethical committee of Jinan University. Because the data were analyzed anonymously, and no apparent ethical research complication with participation could be identified, informed oral consent was recommended and obtained from participants before data collection. Participants were given the opportunity to refuse to participate, to omit questions or to withdraw from the study at any time without penalization.

## Author Contributions

GX and XW participated in the design of this study and they both performed the statistical analysis. XW constructed the overall framework of the study and modified and polished it. GX carried out the study, collected important background information, and drafted the manuscript. AL provided assistance for data acquisition. All authors read and approved the final manuscript.

## Conflict of Interest Statement

The authors declare that the research was conducted in the absence of any commercial or financial relationships that could be construed as a potential conflict of interest.
